# Trust, Information Appraisal, and Health Communication Behaviours Among People Living with Multiple Sclerosis

**DOI:** 10.3390/healthcare14182927

**Published:** 2026-09-09

**Authors:** Adel S. Alhlayl, Haitham Alzghaibi

**Affiliations:** 1 Department of Pharmaceutical Care Services, King Salman Specialist Hospital, Hail Health Cluster, Hail 55471, Saudi Arabia; aalhlayl@moh.gov.sa or adel.alhlayl@uoh.edu.sa; 2 Department of Health Informatics, College of Public Health and Health Informatics, University of Hail, Hail 55471, Saudi Arabia; 3 Department of Health Informatics, College of Applied Medical Sciences, Qassim University, Buraydah 52571, Saudi Arabia

**Keywords:** multiple sclerosis, eHealth literacy, online health information, information quality, health information-seeking, patient empowerment, digital health, United Kingdom

## Abstract

**Background:** People living with multiple sclerosis (MS) are increasingly turning to the Internet for information about their condition and its treatment. However, online health information varies widely in quality, and patients often lack the eHealth literacy needed to distinguish reliable from unreliable sources. **Objective:** This study examined how people with MS in the United Kingdom seek, evaluate, and judge the quality of online health information, with particular attention to information about medicines, and explored the features that they would value in a curated, quality-assessed information resource. **Methods:** A cross-sectional online survey was distributed via the MS Trust to adults with MS or their carers in the United Kingdom. The 55-item instrument, adapted from a previously validated questionnaire, captured demographic characteristics, Internet use, eHealth confidence, perceived quality indicators, assessment difficulties, and preferences for a curated information resource. Descriptive statistics summarised the sample, and Spearman’s rank-order correlation tested associations between confidence, perceived importance of quality, and information-checking behaviours. **Results:** One hundred and fifty-two participants completed the survey. Because the number of individuals reached through the MS Trust’s distribution channels was not recorded, a true response rate could not be calculated; the achieved sample represented approximately 38–40% of the a priori target of 400. Almost all participants (99%) used the Internet to find MS-related information, and 84% sought information about their medicines online. Recommendation by a healthcare professional was the strongest indicator of perceived information quality (17.8%), and MS specialists were the most trusted source overall (32.9%). However, 54.4% of participants expressed concerns about online information quality, and only 47.9% believed that search engines reliably returned high-quality websites. Confidence in evaluating online medicine information correlated strongly and positively with the perceived importance of information quality (ρ = 0.869, *p* < 0.001, *n* = 73) and with active checking behaviour (ρ = 0.677, *p* < 0.001, *n* = 73). Eighty-eight percent of participants endorsed the development of a single, quality-assessed website for MS-related medicine information, and 80% wished for visible details of how each source had been assessed. **Conclusions:** Despite high engagement with online information, people with MS remain uncertain about its quality and find independent assessment time-consuming and complex. A curated, transparently assessed information resource, ideally with clinician endorsement and visible quality indicators, was endorsed by the subset of participants who reached these items and represents a candidate direction for future digital health interventions in MS care. Because several preference items were answered by small subgroups (*n* = 35–79) drawn from an engaged charity membership, these findings are exploratory and require confirmation in more representative samples.

## 1. Introduction

Multiple sclerosis (MS) is a chronic, immune-mediated demyelinating disease of the central nervous system and a leading cause of non-traumatic neurological disability among young and middle-aged adults [1,2]. The third edition of the Atlas of MS reported approximately 2.9 million people living with MS worldwide in 2023, with the United Kingdom among the countries with the highest prevalence at an estimated 158 cases per 100,000 population [1,3,4]. A recent analysis of English primary care records demonstrated that the recorded prevalence more than doubled between 2000 and 2020, with current estimates suggesting that approximately 150,000–190,000 people are living with MS in the UK [5]. The disease typically has its onset in early adulthood, and its episodic and progressive course imposes a substantial and lifelong burden on patients, carers, and health systems [6].

The contemporary management of MS combines disease-modifying therapies, symptomatic medications, rehabilitation, and lifestyle modification [7]. Because management is complex, individualised, and frequently revised over the course of the disease, patient education and self-management have become central to high-quality MS care. Informed patients are better able to participate in shared decision-making, adhere to treatment regimens, and recognise warning signs that warrant clinical review [8,9]. Conversely, knowledge gaps and exposure to inaccurate information have been linked to non-adherence, delayed help-seeking, and reduced quality of life [10].

Against this backdrop, the Internet has become a primary channel through which people with MS acquire health information. A 2023 systematic review of online information-seeking among people with MS reported that between 53.8% and 82% of participants across the included studies used the Internet to look for MS-related content, with searches commonly focused on disease understanding, symptoms, and treatment options [11]. Qualitative work published in 2024 further showed that patients consult online sources not only as a substitute for but increasingly as a supplement to consultations with healthcare professionals, often before and after clinical encounters [12]. The proliferation of disease-specific platforms, social media communities, and patient charity websites has reinforced this trend, and the COVID-19 pandemic accelerated the shift toward digitally mediated health information [13].

### 1.1. Quality of Online Health Information

The democratisation of health information through the Internet has been accompanied by long-standing concerns about quality, accuracy, and the absence of editorial oversight [14]. Online health content is rarely peer-reviewed, frequently lacks transparent authorship, and competes with deliberately misleading or commercially motivated material [15]. The COVID-19 pandemic crystallised this challenge when the World Health Organization described an accompanying “infodemic” of medical misinformation, prompting renewed calls for an “eHealth literacy revolution” [16]. For chronic disease populations such as people with MS, the consequences of acting on inaccurate information are not trivial: documented harms include the inappropriate use of unproven therapies, the abandonment of evidence-based treatment, and psychological distress arising from cyberchondria [17,18].

Several validated instruments have been developed to assess website quality, including DISCERN, the HON Code, the JAMA benchmarks, and, more recently, the WebMedQual scale [19,20]. However, the use of such instruments is rarely transparent to lay users, and survey evidence indicates that few patients examine the “About Us”, authorship, or date-of-update sections of a website before relying on its content [21]. The credibility of a source is therefore frequently inferred from heuristic cues, such as search engine ranking, recommendation by a clinician or peer, or the visual design of a site, rather than from systematic evaluation [22,23].

### 1.2. Theoretical Framework

The present study is anchored in Norman and Skinner’s eHealth literacy framework, the dominant conceptual model in the field of consumer online health information research [24]. The framework positions eHealth literacy as “the ability to seek, find, understand, and appraise health information from electronic sources, and apply that knowledge to addressing or solving a health problem”. Crucially, the model is constructed as the convergence of six distinct but interrelated foundational literacies: traditional literacy (reading and numeracy), information literacy (locating and using information resources), media literacy (critical evaluation of media content), computer literacy (operational ability to use digital devices), scientific literacy (understanding of scientific processes and evidence), and health literacy (understanding and acting on health information). Together, these literacies enable a person to interact competently and critically with online health content.

This framework is particularly well suited to the population of interest in the present study. People living with MS are, by virtue of their chronic condition, repeatedly required to evaluate complex clinical information across the course of the disease—about disease-modifying therapies, symptomatic treatments, lifestyle interventions, and emerging research findings. Recent disease-specific evidence has demonstrated that eHealth literacy is unevenly distributed within the MS population. Tosun and colleagues [25] reported substantial variation in digital appraisal skills among Turkish people with MS, with older age and lower educational attainment associated with weaker eHealth competencies, and Sippel and colleagues [26] documented similarly variable perceptions of usefulness and trust in online sources among German people with MS through their validated eHealth Impact Questionnaire.

**Figure 1:**
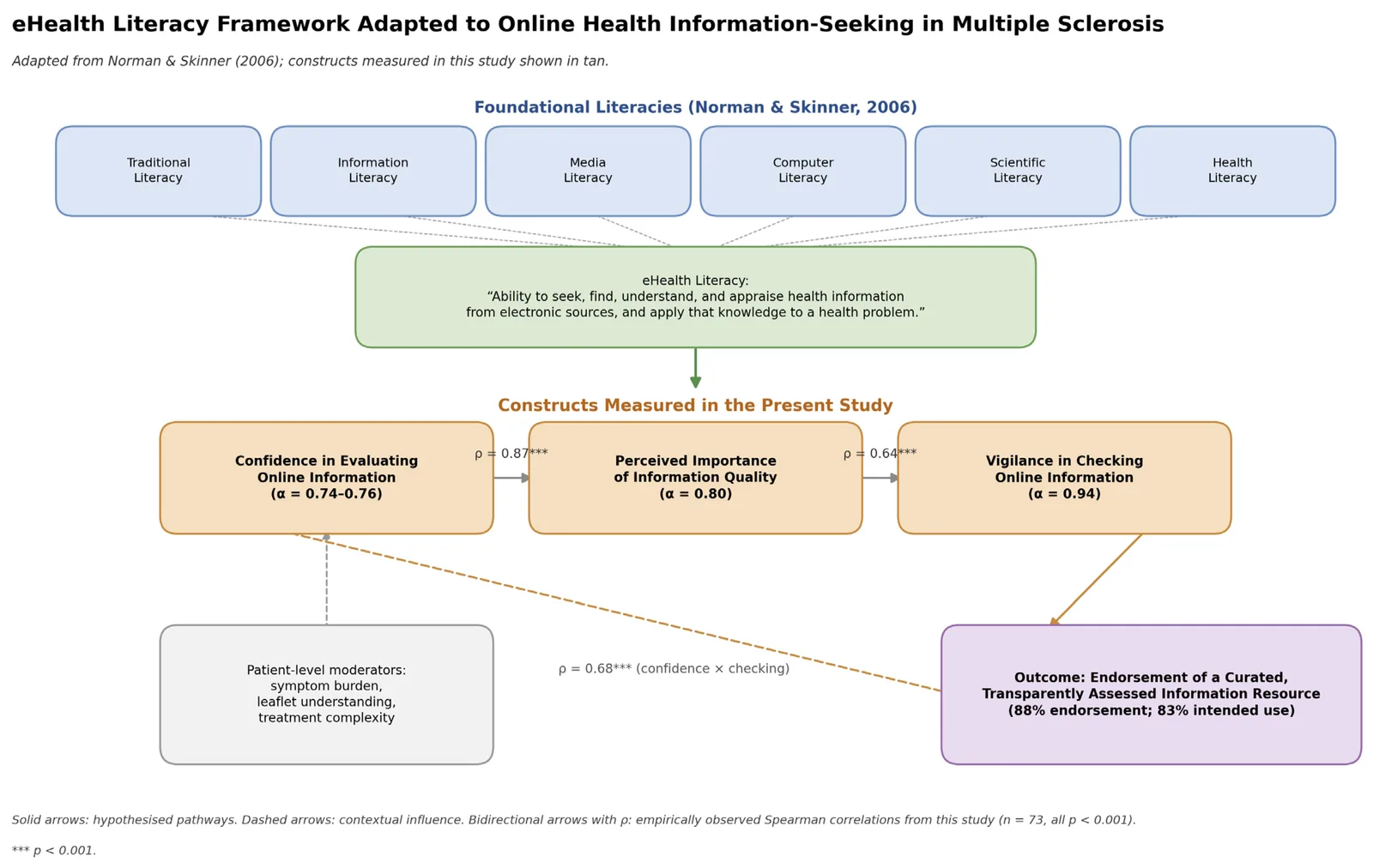
Conceptual framework guiding the present study, adapted from Norman and Skinner [24]. Solid arrows denote hypothesised pathways; dashed arrows denote contextual influence; values with ρ denote empirically observed Spearman correlations from this study (*n* = 73, all *p* < 0.001).

Within Norman and Skinner’s framework, the present study operationalises three core constructs that map onto the analytical appraisal dimensions of eHealth literacy: (i) confidence in evaluating online information (the user’s subjective competence in critically appraising web-based content); (ii) the perceived importance of information quality (the user’s normative valuation of source attributes such as completeness, balance, referencing, and the absence of commercial bias); and (iii) vigilance in checking online information (the user’s actual reported behaviour of inspecting authorship, currency, accuracy, and the factual basis). Within Norman and Skinner’s six foundational literacies, confidence in evaluating online information draws primarily on media and computer literacy; the perceived importance of information quality reflects information and scientific literacy; and vigilance in checking operationalises the analytical convergence of information, media, and health literacy. These three constructs are theorised to be positively interrelated and to jointly predict patient receptivity to externally curated information resources. To our knowledge, this is the first study to combine a UK-specific MS sample, an explicit focus on information about medicines, and the direct elicitation of resource feature preferences within a single eHealth literacy framework, extending prior work that has treated these elements separately. The full conceptual framework, including the hypothesised pathways and the empirically observed associations from this study, is presented in Figure 1.

Two additional theoretical observations follow from this framing. First, the framework predicts that patient-level moderators such as the disease symptom burden, treatment complexity, and difficulty understanding patient information leaflets will exert contextual influences on the demand for, and willingness to invest effort in, online information appraisal. Second, where the appraisal demands of independent evaluation exceed a patient’s eHealth literacy resources, theory predicts a corresponding increase in receptivity to externally curated information sources. The latter prediction provides the rationale for the present study’s primary outcome of interest, namely participant endorsement of a single curated and transparently assessed information resource.

### 1.3. Information About Medicines

Information about medicines occupies a particularly sensitive position within MS-related online content. Disease-modifying therapies for MS are complex, often costly, and associated with non-trivial risk–benefit trade-offs that patients are increasingly expected to weigh in shared decision-making [27]. Patient information leaflets included with medicines are mandated by regulation but are widely reported to be difficult to read and to satisfy patients’ information needs poorly [28]. Survey evidence has long suggested that, when leaflets fail to meet expectations, patients turn to the Internet, where the quality of information about specific medicines is heterogeneous and often poor [29,30,31]. Investigations of online pharmacies have additionally documented the availability of prescription-only medicines without adequate prescribing information, and isolated cases of harm associated with online medicine purchase have been reported in the peer-reviewed literature [32,33].

In the United Kingdom, this landscape is shaped by a specific regulatory and information architecture. The National Health Service (NHS) provides a widely used public information portal (NHS.uk), while the Medicines and Healthcare Products Regulatory Agency (MHRA) regulates the licensing, safety, and advertising of medicines and operates the Yellow Card scheme for adverse event reporting. Registered online pharmacies must display the General Pharmaceutical Council and MHRA distance selling logos. Despite this framework, patients navigating search results are rarely able to distinguish regulated from unregulated sources at the point of use, which motivates interest in curated, transparently assessed resources.

### 1.4. Gap in the Evidence and Rationale for the Present Study

Despite the growing literature on online health information-seeking, comparatively few studies have examined how people with MS specifically judge the quality of what they find, the difficulties that they encounter when attempting to evaluate online content, and which features they would value in a curated, quality-assessed resource. The 2023 systematic review by Berhanu and colleagues explicitly identified this as a research gap and recommended that “quality sources of online information should be accessible to people with MS”, with clinician-mediated signposting [11]. The present study addresses this gap by characterising the information-seeking behaviour, perceived quality cues, and assessment difficulties reported by a UK sample of people with MS, with a particular focus on information about medicines, and by interpreting these findings within the eHealth literacy framework described above.

### 1.5. Study Aim and Objectives

The aim of this study was to characterise the perceptions and behaviours of people living with MS in the United Kingdom regarding the quality of online health information, with a specific focus on information about medicines. The specific objectives were as follows:

1. To describe how people with MS use the Internet to find MS-related information and information about their medicines;
2. To explore how people with MS judge the quality of the online information that they find;
3. To identify the difficulties that patients encounter when attempting to assess online information quality;
4. To explore the features that people with MS would value in an online tool or curated resource intended to help them to identify good-quality information about MS-related medicines.

## 2. Materials and Methods

### 2.1. Study Design

This study employed a cross-sectional exploratory design using a structured, self-administered online questionnaire. The reporting of this study follows the Checklist for Reporting Results of Internet E-Surveys (CHERRIES) [34] and the Strengthening the Reporting of Observational Studies in Epidemiology (STROBE) cross-sectional guidance [35].

### 2.2. Setting and Participants

The survey was distributed online via the MS Trust, a national UK charity that provides information and support to people living with MS and maintains direct communication channels with patients across the United Kingdom. Eligible participants were adults (aged 18 years or older), residing in the UK, with a confirmed diagnosis of MS, or carers responding on behalf of a person with MS. Participants were required to be able to read English and to have Internet access to complete the survey.

The MS Trust reaches a self-selected membership of people with MS and carers who have actively chosen to engage with a patient support charity; this membership is likely to be more digitally engaged, more proactive in information-seeking, and better resourced than the general UK MS population—a point that is returned to in Section 4.7 Precise membership demographics are not published by the charity, which constrains formal comparison with national MS registry figures.

### 2.3. Sample Size and Sampling Strategy

A convenience sampling strategy was adopted given the absence of a sampling frame that would have permitted random selection. The target sample size was calculated using Yamane’s formula at a 5% margin of error and 95% confidence level, taking the UK MS population as approximately 150,000–190,000. Yamane’s formula was chosen because it gives a transparent, widely used, closed-form estimate that is stable for large populations: above roughly 100,000, the required sample is effectively insensitive to the exact population size, so the same target of approximately 384–400 is obtained whether the UK MS population is taken as 100,000 or the current higher estimate of 150,000–190,000. The lower figure was retained only as the conservative input to the original calculation and did not alter the target [36], which was 400 participants. This target was consistent with the value of *n* = 384 obtained from the Krejcie and Morgan sample size table for a population of this magnitude [37]. The achieved sample was 152 participants. Because participants were recruited by convenience rather than probability sampling, a conventional margin of error does not strictly apply; the sample size target is therefore best read as an indication of the precision achievable under ideal sampling, and all estimates should be interpreted as exploratory, with correspondingly limited precision. Because the MS Trust distributed the survey through open communication channels rather than to an enumerated invitation list, the total number of individuals who received or viewed the invitation was not captured, so a conventional response rate cannot be computed; for transparency, we report only that the achieved sample corresponded to approximately 38–40% of the a priori target of 400. This achieved sample size is comparable to those of other UK MS questionnaire studies distributed through charities and clinics [38,39].

### 2.4. Survey Instrument

The questionnaire was adapted, with written permission, from an instrument originally developed and validated by Nicolson in a doctoral thesis examining leaflet-based and Internet-based information about medicines for consumers [40]. Nicolson’s instrument was originally developed for a general medicine information population; adaptation for the present study involved (i) reframing item stems so that they could be answered by people with MS or carers acting on their behalf; (ii) adding MS-specific content on the disease subtype, symptom burden, and disease-modifying therapies; (iii) inserting a dedicated block on preferences for a curated MS medicines resource; and (iv) removing items not relevant to a chronic neurological population. The adapted instrument was reviewed for content and face validity by the research team, which included health informatics and pharmacy expertise, and by a small number of people with lived experience of MS. It then underwent a formal pilot with a convenience sample of pilot respondents, after which minor wording ambiguities were clarified, two double-barrelled items were split, and the estimated completion time was confirmed; pilot responses were not included in the analytic dataset. The full instrument is provided as Supplementary Material Table S1. The final instrument comprised 55 closed-ended items, with selected open-text fields, organised into the following sections: (i) demographic and background characteristics; (ii) frequency and methods of Internet use for MS-related information; (iii) confidence in evaluating online information about MS; (iv) perceived indicators of, and barriers to assessing, online quality; (v) preferences for a single curated information resource and its features; (vi) information-seeking specifically about medicines; (vii) confidence in evaluating online medicine information; and (viii) vigilance in checking online information quality (a 7-point Likert frequency scale). Items were structured to operationalise the eHealth literacy constructs described in Section 1.2. The estimated completion time was 20–30 min.

**Scale construction and scoring.** Composite variables were constructed as follows. Confidence in evaluating the quality of online medicine information, perceived importance of information quality, vigilance in checking, and readiness of medicine information were each scored as the sum of their constituent Likert items (higher totals indicating greater confidence, importance, vigilance, or readiness) and are reported as total scores. Total MS symptoms was computed as the count of endorsed items across the 16-symptom checklist (each scored 0 = absent, 1 = present; possible range 0–16), and total treatments received was computed as the count across the eight-item treatment list (each 0 = no, 1 = yes; range 0–8). Understanding of the patient information leaflet and review of prescribed medicines were single items scored on a 1–5 scale. Where a multi-item scale had missing responses, the scale total was computed from the available items for participants who completed the relevant section; no imputation was performed.

### 2.5. Data Collection Procedure

The finalised survey was hosted on SurveyMonkey and distributed by the MS Trust to its registered members through the trust’s established communication channels, comprising its email newsletter to registered members and posts on its official social media accounts. Data were collected between January and March 2026. Because distribution combined email and social media channels, mode effects could not be fully excluded. Participation was anonymous and voluntary. The first page of the survey contained the participant information sheet, and informed consent was obtained electronically before the participant could proceed to the survey items. Participants were informed that they could withdraw at any point by closing the browser, in which case no data would be transmitted; once submitted, anonymous responses could not be identified for later withdrawal. Participants who reported concerns or distress arising from any item were directed to their general practitioner or local MS support service.

### 2.6. Ethical Considerations

Ethical approval for this study was granted by the College of Health and Human Sciences and Medical School Research Ethics Committees of Swansea University (reference: 120716-536264). The research was conducted in accordance with the UK Policy Framework for Health and Social Care Research and the principles of the Declaration of Helsinki. All participants provided informed consent electronically prior to participation.

### 2.7. Statistical Analysis

Data were exported from SurveyMonkey to IBM SPSS Statistics version 25 (IBM Corp., Armonk, NY, USA). Continuous variables were summarised as the mean and standard deviation (SD) and categorical variables as frequencies and percentages. Where the denominator for a question varied because of skip logic or non-response, the valid denominator is reported alongside each result. The distributional properties of ordinal Likert items were inspected and judged non-normal; non-parametric Spearman’s rank-order correlation (ρ) was therefore used to examine associations between key composite scales (confidence in evaluating online medicine information; perceived importance of information quality; vigilance in checking; readiness of medicine information; total reported symptoms; total reported treatments; and understanding of the patient information leaflet). Internal reliability was assessed for each multi-item scale using Cronbach’s alpha. A two-tailed *p*-value of less than 0.05 was treated as statistically significant. Because multiple bivariate correlations were tested, *p*-values for the correlation matrix were additionally adjusted for multiple comparisons using the Benjamini–Hochberg false discovery rate (FDR) procedure, and both unadjusted and adjusted values are reported. Ninety-five percent confidence intervals for the correlation coefficients were derived by Fisher z-transformation, using the Bonett–Wright standard error appropriate for Spearman’s ρ; 95% confidence intervals for Cronbach’s alpha were obtained using the Feldt method. Multicollinearity among the composite scales was examined because several inter-scale correlations were high; scales correlating at ρ > 0.80 are flagged and interpreted as potentially overlapping rather than fully distinct constructs. As the data are cross-sectional and entirely self-reported, associations may be inflated by common method bias, which is acknowledged in the interpretation. Selected open-text fields were reviewed to contextualise the closed-item findings but, given their limited volume, were not subjected to formal qualitative coding; they are reported only where they illustrate a closed-item result. Missing data were handled by pairwise deletion, and the valid denominator is reported transparently for each analysis. Because non-response was concentrated in later survey sections (indicating dropout rather than item-specific refusal), results based on reduced denominators are flagged in the text. All analyses were conducted on the combined sample of people with MS and carers. To address the possibility that patients and carers differed in their responses, we performed a sensitivity analysis restricted to individuals with MS only (*n* = 62 for the medicine–confidence/importance scales; *n* = 79 for the readiness scales). The results of this sensitivity analysis are reported in Supplementary Table S1 and summarised in Section 4.7. The primary correlational results reported in Table 2 and Figure 5 are based on the combined sample, with the patient-only sensitivity results interpreted as exploratory due to the smaller sample size.

## 3. Results

### 3.1. Sample Characteristics

One hundred and fifty-two individuals participated in the study. Of these, 128 (84.2%) reported being a person with MS, 22 (14.5%) reported being a carer, and the respondent type was missing for two participants (1.3%). Percentages for each variable were calculated against its valid (non-missing) denominator, so category percentages may sum to slightly less than 100% where responses were missing and may differ from 100% by ±0.1% because of rounding; the number of missing responses per variable is reported in Table 1. The sample was predominantly female (68.3%) and skewed towards older age groups, with 74.8% of respondents aged 46 years or above. Educational attainment was relatively high: 27.3% reported postgraduate qualifications, 30.1% an undergraduate degree, and 25.9% A-level or equivalent qualifications. Almost 70% of respondents were in employment (full-time, part-time, or self-employed), and the most commonly reported MS subtypes were relapsing–remitting MS (37.8%) and secondary progressive MS (34.3%). The mean age at MS diagnosis was 45.5 years (SD = 14.13). Detailed demographics are presented in Table 1.

### 3.2. Use of the Internet for MS-Related Information

Internet use for MS-related information was almost universal in the sample. Of 145 participants who responded to this item, 99.3% reported using the Internet to find MS information, with frequencies distributed as daily (18.6%), weekly (27.6%), as needed (46.9%), or monthly (6.2%). Only one participant (0.7%) reported never using the Internet for MS information.

### 3.3. Confidence in Evaluating Online MS Information

**Figure 2:**
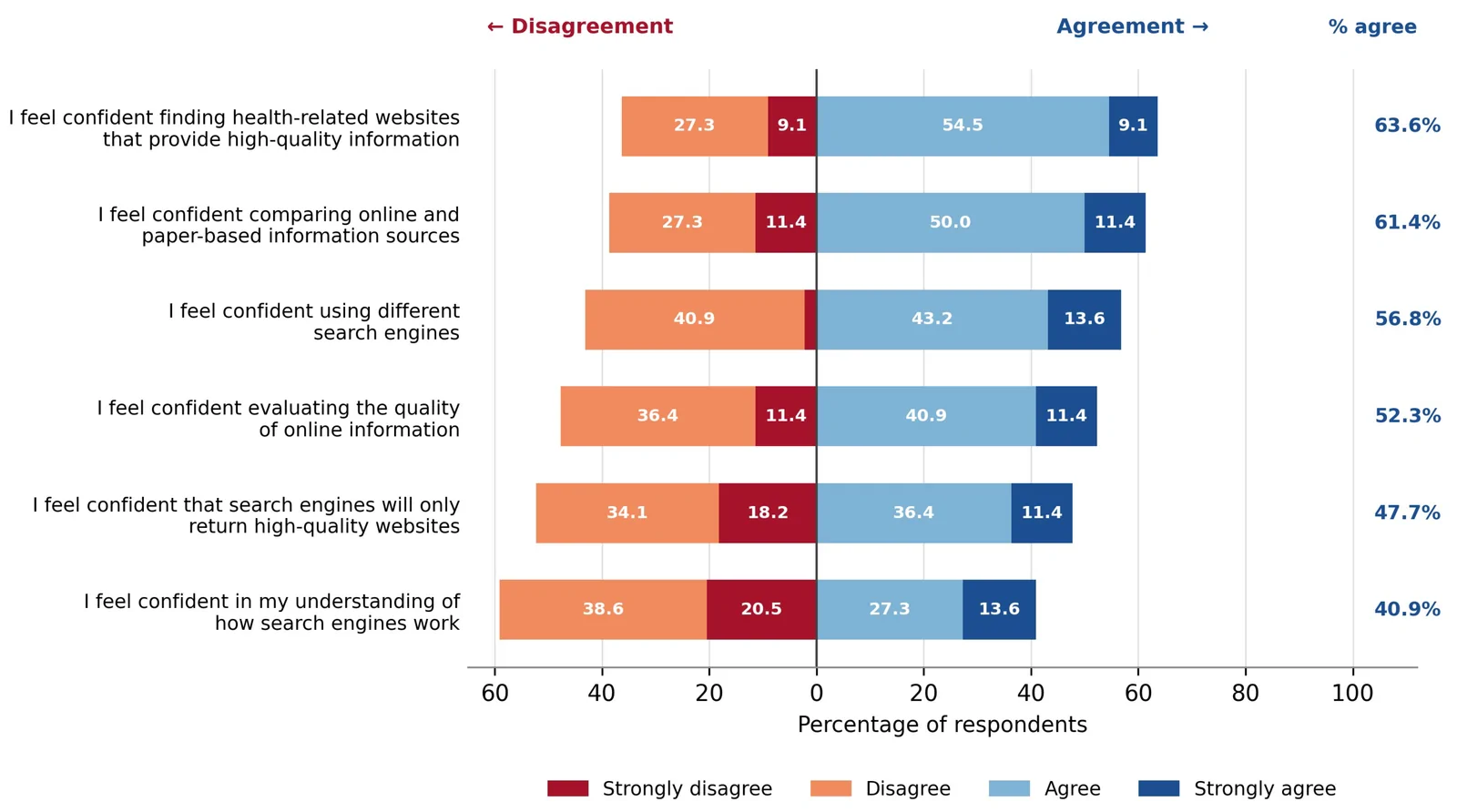
Diverging stacked bar chart of participants’ confidence in evaluating the quality of online MS information (*n* = 44). Items are ordered by total agreement (highest at top). Total agreement (%) is shown on the right.

Confidence in evaluating the quality of online MS information was assessed using a six-item scale (Cronbach’s α = 0.76). Overall confidence was modest but uneven across items (Figure 2). Confidence was highest for the ability to find health-related websites that provided high-quality information (63.6% agreement) and to compare sources across online and paper-based media (61.3%). Confidence was lowest for understanding how search engines work (41.0%) and for trusting search engines to return only high-quality websites (46.5%), indicating a substantial gap between participants’ perceived ability to find information and their confidence in the algorithmic mechanisms that surface it. The reduced denominator for this item block (*n* = 44) reflects survey dropout and skip logic rather than item-specific refusal: this section appeared later in a 20–30 min instrument, and completion declined across successive sections. Findings from this block should therefore be read as indicative of the subsample that persisted to it rather than of the full sample of 152.

### 3.4. Perceived Indicators of Information Quality

**Figure 3:**
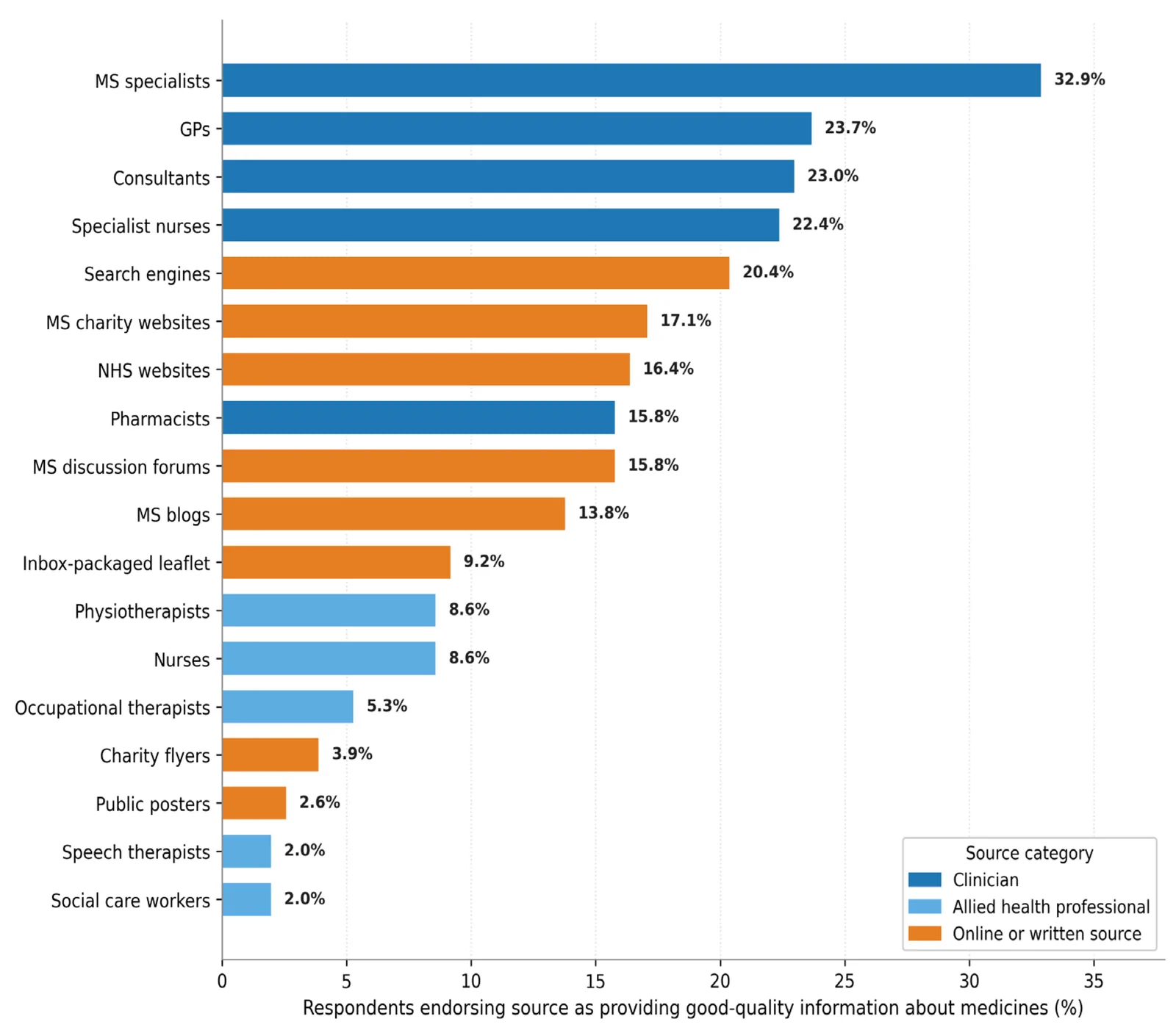
Sources of information about medicines ranked by perceived quality (*n* = 152), coloured by source category. Clinicians dominated the top of the ranking, with allied health professionals concentrated at the bottom and online/written sources distributed across the middle of the range.

When asked which cues led them to judge that online information was of good quality, recommendation by a healthcare professional was the most frequently endorsed indicator (17.8%), followed by media advertisement (13.8%) and links from a recognised website or blog (13.2%); recommendation by a friend was endorsed by 7.9%. Notably, 3.9% of respondents reported that they did not know what indicated good-quality information. Because respondents could endorse more than one indicator and could also leave the item unanswered, these percentages are proportions of all endorsements rather than a mutually exclusive distribution and do not sum to 100%; the remaining proportion corresponds to unselected options and non-response. They should therefore be read as relative endorsement rather than as a partition of the sample. When asked separately about the most trusted sources of medicine-related information, MS specialists were the most commonly endorsed source (32.9%), followed by general practitioners (23.7%), consultants (23.0%), and specialist nurses (22.4%). The full ranking of all 18 information sources, grouped by source category (clinician, allied health professional, online or written source), is presented in Figure 3.

### 3.5. Difficulties Assessing Online Information Quality

Participants reported several barriers to independently assessing online information quality. Within general MS information, 16.4% considered quality assessment too complicated, 14.5% reported that it took too much time, and 14.5% reported not knowing how to assess quality. When asked specifically about online medicine information, these difficulties were more pronounced: 29.6% reported that assessment took too much time, 21.7% did not know how to assess quality, 19.1% considered assessment too complicated, and 17.8% had never been trained on how to assess information quality.

### 3.6. Preferences for a Single Curated Information Resource

**Figure 4:**
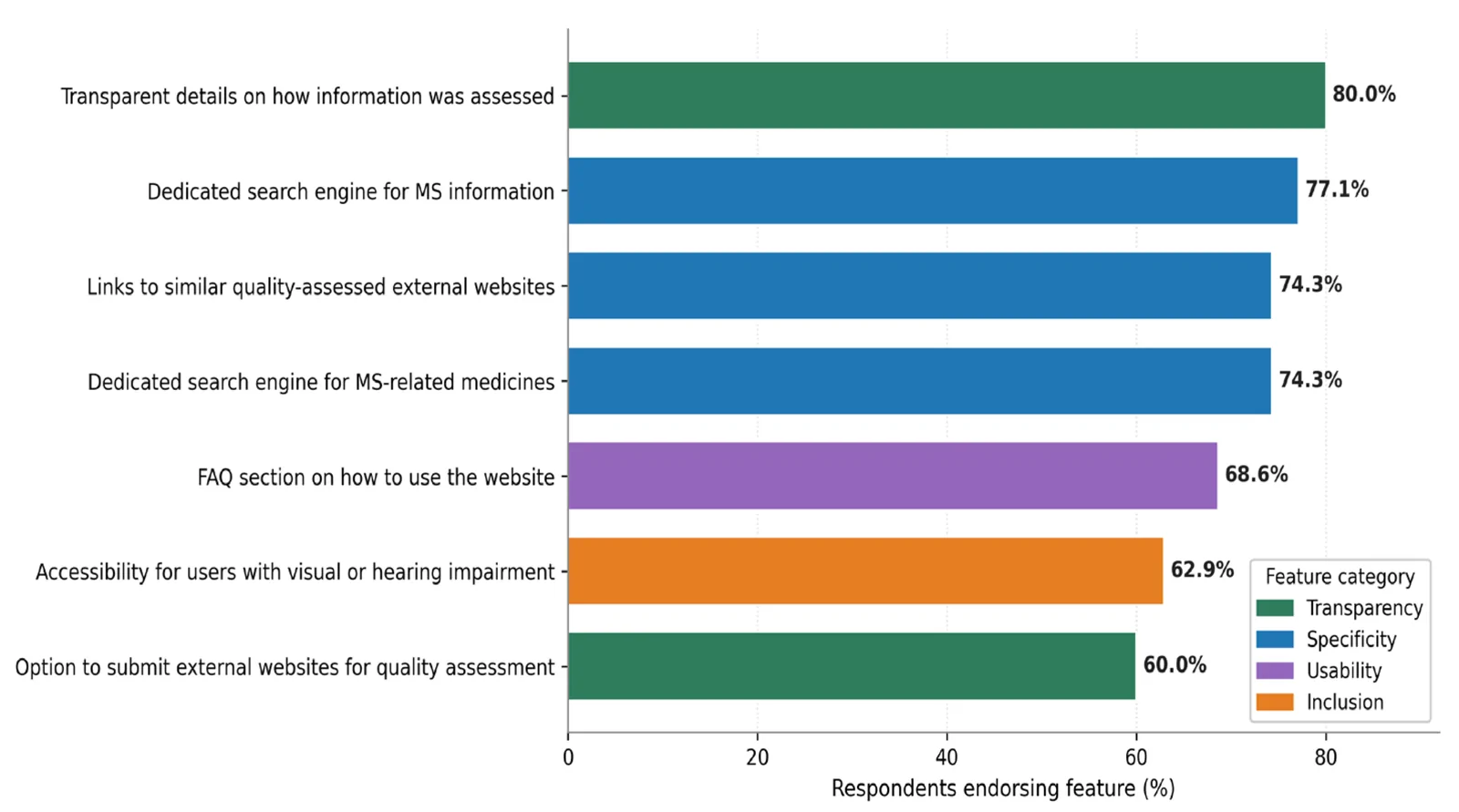
Preferred features of a curated information resource for MS-related medicines (*n* = 35), grouped by feature category. Features related to transparency of quality assessment and to MS-specific search functionality received the highest endorsement, while accessibility for users with sensory impairment was endorsed by almost two-thirds of respondents.

There was strong support among participants for a single, curated information resource that presented only quality-assessed information. For MS information generally, 79.5% of respondents who answered this item (*n* = 44) endorsed a single curated website, and, for medicine information specifically, 87.7% of respondents (*n* = 73) considered such a resource helpful, with 82.9% (*n* = 35) reporting that they would use it to inform their decisions if it were available. These denominators differ because the items appeared in successive survey sections affected by progressive dropout: the *n* = 35 who answered the decision use item were a subset of the *n* = 73 who answered the medicine resource item, who were in turn largely a subset of earlier respondents. The small denominators (*n* = 35–73) mean that these preference estimates are drawn from the most persistent respondents and may not generalise to the full sample; they are reported here as exploratory. The preferred features of such a resource clustered around four themes: transparency of quality assessment, specificity to MS and medicines, usability, and inclusion (Figure 4).

With respect to how quality assessment results should be displayed, no single format dominated: a percentage quality score next to each result was the modal preference (16.4%), followed equally by star ratings (15.8%) and a traffic light system (15.8%), with 9.9% favouring standard search results annotated with quality marks.

### 3.7. Online Information About Medicines and Vigilance in Checking

Most participants used the Internet to seek information about their medicines, and 67.4% reported actively preferring to obtain such information online. Health professionals remained the most strongly preferred source overall (92.5% agreement), with leaflets supplied with medicines being the least preferred (54.3%). Importantly, 54.4% of respondents (*n* = 79) reported concerns about the quality of online medicine information.

Vigilance in checking specific quality features of online medicine information was assessed using a seven-item scale (Cronbach’s α = 0.94). Frequent checking (defined here as responses of “frequently”, “usually”, or “all the time” on a seven-point scale) varied substantially by item: accuracy (49.3%) and whether information was fact or opinion (49.3%) were checked most often, followed by comprehensiveness (45.2%), the author’s goals and objectives (44.4%), whether information was up to date (41.1%), the identity of the author (32.9%), and contact information for the author (28.8%).

The perceived importance of information quality was assessed using a four-item scale (Cronbach’s α = 0.80). Most participants endorsed information completeness (87.5%), accurate referencing (79.2%), the absence of commercial promotion (70.8%), and balanced presentation (65.3%) as important quality features.

### 3.8. Associations Between Confidence, Perceived Quality, and Checking Behaviour

**Figure 5:**
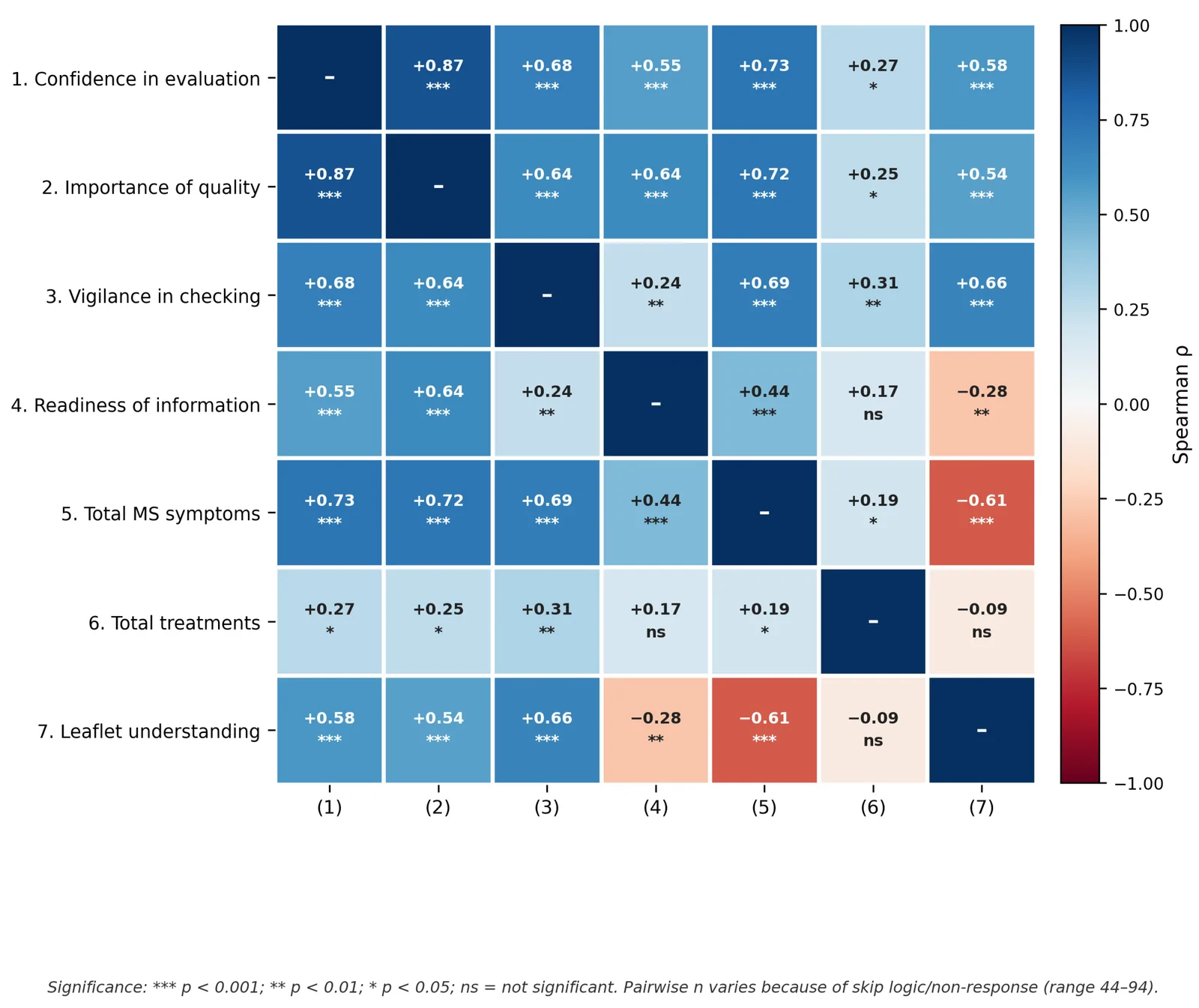
Spearman rank-order correlation matrix between key constructs measured in the study. Blue cells indicate positive correlations; red cells indicate negative correlations; intensity of colour reflects magnitude. Significance markers use distinct symbols.

To explore the structure of the relationships between these constructs, Spearman’s rank-order correlations were computed between confidence in evaluating online medicine information, perceived importance of information quality, vigilance in checking, readiness of medicine information, total symptoms of MS, total treatments received, and understanding of the patient information leaflet (Figure 5; Table 2). All correlations were computed on the combined sample of people with MS and carers. A patient-only sensitivity analysis was performed and is reported in Supplementary Table S1 and summarised in Section 4.7.

Confidence in evaluating online medicine information showed a strong positive correlation with perceived importance of information quality (ρ = 0.869, *p* < 0.001) and with vigilance in checking (ρ = 0.677, *p* < 0.001). Confidence also correlated positively with readiness of medicine information (ρ = 0.553, *p* < 0.001), total symptoms of MS (ρ = 0.731, *p* < 0.001), total treatments received (ρ = 0.273, *p* < 0.05), and understanding of the patient information leaflet (ρ = 0.583, *p* < 0.001). Perceived importance of information quality was likewise correlated with vigilance in checking (ρ = 0.641, *p* < 0.001), readiness (ρ = 0.636, *p* < 0.001), symptom burden (ρ = 0.722, *p* < 0.001), and leaflet understanding (ρ = 0.536, *p* < 0.001). Readiness of medicine information correlated positively with symptom burden (ρ = 0.437, *p* < 0.001) and negatively with leaflet understanding (ρ = −0.279, *p* < 0.01), suggesting that patients with greater symptom burdens and poorer leaflet understanding place a higher premium on rapid access to online medicine information. The very strong association between confidence and perceived importance of quality (ρ = 0.869) approaches conventional multicollinearity thresholds and indicates that, empirically, these two constructs overlap substantially; they may reflect a single underlying analytical appraisal factor rather than two independent dimensions (see Section 4). All correlation analyses were conducted on the full combined sample of people with MS and carers (*n* = 73 for medicine–confidence/importance scales; *n* = 94 for readiness scales). A sensitivity analysis restricted to people with MS only was not performed because the small pairwise denominators (*n* = 73 for the key scales) would have yielded unstable estimates when further restricted to the patient subset. The results should therefore be interpreted as reflecting the combined sample of patients and carers.

## 4. Discussion

This study set out to characterise how people living with MS in the United Kingdom seek and evaluate online health information, with particular attention to information about medicines, and to elicit their preferences for a curated, quality-assessed information resource. The findings are interpreted below within the eHealth literacy framework introduced in Section 1.2, with each of four substantive themes discussed in turn.

### 4.1. Online Information-Seeking Is Now Near-Universal Among People with MS

Nearly all participants (99%) used the Internet to find MS-related information, and 84% sought information about their medicines online. This is substantially higher than the 63% reported by Lejbkowicz and colleagues in 2010 [41] and is at the upper end of the range (53.8–82%) summarised by the 2023 systematic review of Berhanu et al. [11]. Two trends help to explain this shift. First, Internet penetration and digital literacy have advanced considerably over the intervening period, particularly among older adults, who constitute a large proportion of the UK MS population [42]. Second, the COVID-19 pandemic accelerated digital health-seeking and normalised the use of online sources for clinical content [13]. These comparisons should, however, be read with caution. The present sample was drawn from an engaged patient charity and is likely to over-represent digitally active, information-seeking individuals, so part of the apparent increase over earlier studies such as Lejbkowicz et al. [41] may reflect differences in sampling frame rather than a genuine secular trend. Similarly, while the COVID-19 pandemic plausibly accelerated digital health-seeking, the present cross-sectional design and the fact that the exact data collection period is not reported here mean that we cannot attribute the high engagement specifically to the pandemic; this remains a contextual hypothesis rather than a demonstrated effect. With these caveats, our findings are consistent with the view that any contemporary discussion of MS patient education should take online information-seeking as a baseline expectation rather than a marginal behaviour.

### 4.2. Trust Is Selective and Anchored in Clinician Recommendation

Despite high engagement with online sources, participants placed substantially greater trust in clinicians than in any web-based or media-based cue. As shown in Figure 3, recommendation by a healthcare professional was the strongest indicator of perceived information quality (17.8%), and MS specialists, general practitioners, consultants, and specialist nurses were collectively endorsed by a third or more of respondents as trusted sources of medicine information. This pattern aligns closely with the findings of Marrie et al. [43] and the qualitative work of Bevens and colleagues [12], who reported that people with MS treat clinician endorsement as the most reliable proxy for source credibility. Importantly, this does not mean that patients defer to clinicians for information acquisition: the same participants both consulted clinicians and searched the Internet, frequently before and after clinical encounters. This produces an apparent paradox: participants trusted clinicians most as a quality marker, yet the majority (67.4%) actively preferred to obtain medicine information online. The most plausible reading is not distrust of clinicians but that online sources offer convenience, immediacy, and the ability to revisit information at one’s own pace, complementing rather than replacing the consultation; some patients may also perceive time-limited consultations as insufficient for detailed medicine questions. The implication is not that online resources are dispensable but rather that clinician-mediated signposting could materially shape which online resources patients trust and use—a point explicitly recommended by the 2023 systematic review of Berhanu et al. [11].

### 4.3. Independent Quality Assessment Is Effortful, Uncertain, and Selectively Performed

More than half of the respondents (54.4%) reported concerns about the quality of online medicine information, and only 47.9% believed that search engines reliably returned high-quality websites (Figure 2). Despite these concerns, participants reported only partial engagement in quality-checking: approximately half checked information for accuracy and whether content was fact or opinion, but fewer than a third routinely checked author identity, and barely a quarter checked author contact details. This selective vigilance is consistent with the broader eHealth literacy literature, which has demonstrated that quality cues attributable to surface design and authority signals (recommendation, brand) are more salient to lay users than the systematic appraisal of provenance and currency [21,22,23]. Almost a third of the participants (29.6%) reported that the assessment of online medicine information was too time-consuming, and roughly one in five reported either not knowing how to assess quality or never having been trained to do so. Tosun et al. [25] recently documented a similar profile of variable eHealth competencies among Turkish MS patients, suggesting that this is not a phenomenon localised to the UK context.

The strong positive correlation observed between confidence in evaluation, perceived importance of quality, and active checking (ρ = 0.641–0.869, all *p* < 0.001; Figure 5) is theoretically coherent and practically informative. Within the eHealth literacy framework, these three constructs map closely onto Norman and Skinner’s analytical appraisal dimension [24], and their tight covariation suggests that interventions targeting any one of them may yield gains in the others. An important alternative interpretation must be acknowledged: the very high correlation between confidence and perceived importance of quality (ρ = 0.869) may indicate that these are not empirically distinct constructs but rather indicators of a single latent analytical appraisal factor. Because all measures were self-reported in one cross-sectional questionnaire, some of this covariation may also reflect common method bias rather than true construct overlap. Confirmatory factor analysis in a larger sample would be needed to establish whether a one-factor or multi-factor structure better fits these data; the present correlations should be read as hypothesis-generating in this respect. The further finding that confidence in evaluation correlates positively with symptom burden (ρ = 0.731) and with poorer leaflet understanding (ρ = −0.279 between leaflet understanding and readiness) suggests that patients with greater unmet information needs are more strongly motivated to develop appraisal skills but also more vulnerable to the consequences of poor-quality content.

### 4.4. Strong Endorsement of a Single, Transparently Assessed Information Resource

Eighty-eight percent of participants endorsed the development of a single curated website for MS-related medicine information, and 83% indicated that they would use such a resource if it were available. Preferred features (Figure 4) clustered around four themes that are theoretically coherent within the eHealth literacy framework: transparency of quality assessment (80% wished to see how each information source had been assessed; 60% desired the ability to submit external sites for assessment), specificity (74–77% desired dedicated MS and MS medicine search functionalities, with links to similar quality-assessed resources), usability (69% desired a frequently asked questions section), and inclusion (63% desired accessibility features for users with visual or hearing impairments). These preferences are consistent with broader findings from the 2024 PLOS Digital Health systematic review on layperson-facing interventions to identify trustworthy digital health information, which concluded that competence-based and problem-based search learning approaches outperform passive media literacy interventions [44]. The preference for visible, transparent scoring (whether as a percentage, star rating, or traffic light system) suggests that any future tool should expose its evaluative criteria to the user rather than treating quality as a black-box ranking decision. These endorsements should nonetheless be interpreted cautiously. They rest on small denominators (*n* = 35–79) drawn from an engaged charity membership, and high endorsement of an evidently desirable resource is susceptible to social desirability bias; the strength of support may therefore be inflated relative to the broader MS population. Practically, a single curated resource also raises non-trivial challenges regarding cost, governance, ongoing maintenance, and the difficulty of staying current in a fast-moving evidence base, alongside potential unintended consequences such as patients over-relying on one source, reduced independent appraisal skills, or the resource itself becoming outdated or subtly biased. These considerations temper, without negating, the case for such a resource.

### 4.5. Theoretical Implications

Viewed through the lens of the eHealth literacy framework presented in Figure 1, our findings map cleanly onto the model proposed by Norman and Skinner [24]: participants demonstrated reasonably high functional and media literacy (they searched, navigated, and accessed information effectively) but weaker information and analytical literacy (they struggled with appraisal of authorship, currency, and bias). The strong covariation among the three study-measured constructs (confidence, perceived importance, vigilance) supports the framework’s prediction that the analytical appraisal dimensions of eHealth literacy operate as a coherent latent factor rather than as independent skills, and the role of patient-level moderators (symptom burden, leaflet understanding) is consistent with the framework’s expectation that contextual factors shape both the demand for and the perceived burden of online appraisal. This pattern reinforces calls in the recent literature for an “eHealth literacy revolution” in chronic disease populations [16] and for clinician-mediated curation as a feasible mid-term solution while population-level eHealth literacy is being built up [11].

### 4.6. Implications for Practice and Policy

Three practical implications follow. First, MS clinicians, specialist nurses, and patient charities are exceptionally well positioned to act as gatekeepers to high-quality online information, given that recommendation by these groups was the dominant quality cue endorsed by participants. Embedding brief signposting to curated resources within routine MS consultations may be a low-cost intervention, although the present study did not evaluate its effectiveness. Second, the development or endorsement of a single, transparently assessed resource, supported by recognised charities or national health bodies, might reduce the cognitive burden of independent quality assessment for patients who report this burden as a barrier, although whether it would do so was not tested here. Third, accessibility features should not be optional; the prevalence of visual, cognitive, and motor impairment within MS makes universal design a clinical as well as ethical imperative.

### 4.7. Strengths and Limitations

This study has several strengths. The use of a national distribution channel (the MS Trust) yielded a UK-wide sample that included carers as well as patients and covered the full range of MS subtypes. The instrument was adapted from a previously validated questionnaire and underwent a formal pilot with iterative refinement. Internal consistency was acceptable to high across all multi-item scales (α = 0.74–0.94), and inferential analyses using non-parametric methods provided robust insight into the structure of the relationships between key constructs. The study is also, to our knowledge, the first to interpret these findings explicitly within Norman and Skinner’s eHealth literacy framework in a UK MS population.

Several limitations should also be acknowledged. First, the use of convenience sampling through the MS Trust, while pragmatic, may have introduced selection bias toward patients who were more engaged with patient-facing organisations and digital resources; this likely overestimates Internet use and may underestimate barriers in less-engaged subgroups. Because the MS Trust membership probably skews toward patients who are more proactive, better resourced, and more digitally engaged, and because membership demographics (disease severity, socioeconomic status, and geographical spread within the UK) are not published, the sample may not represent the wider UK MS population; the likely direction of this selection bias is to overstate eHealth engagement and appraisal activity. Second, the achieved sample of 152 was below the calculated target of 400, although it remained consistent with sample sizes reported in comparable UK MS studies [38,39]. This has direct implications for statistical power and precision: with pairwise denominators as low as 44–96 for the correlation analyses, coefficients were estimated with wide confidence intervals, and weaker associations may not have been reliably detectable, so point estimates from smaller subsamples should be read as imprecise. Third, skip logic and item non-response produced reduced denominators for several questions (notably the curated website items, *n* = 35–79), and these results should be interpreted accordingly. Fourth, because eligibility required English literacy and Internet access, the findings do not generalise to non-English-speaking people with MS in the UK or to those with limited digital access, who may be among the most vulnerable and in greatest need of good-quality information; their perspectives are, by design, absent here. Fifth, respondents comprised both people with MS (85.3%) and carers (14.7%), who may differ in information-seeking behaviour, quality perceptions, and medicine information needs; the sample was not powered to compare these groups, and pooled estimates may mask such differences. To address this concern, we performed a patient-only sensitivity analysis, restricting the sample to individuals with MS (*n* = 62 for the medicines-confidence/importance scales; *n* = 79 for the readiness scales). As shown in Supplementary Table S1, the direction, magnitude, and statistical significance of all key correlations were highly consistent with those observed in the combined sample, with correlation coefficients differing by no more than 0.02–0.03. This provides reassurance that the inclusion of carers did not materially distort the primary findings. However, because the patient-only subsample was smaller (*n* = 62–79), these results should be interpreted as exploratory, and formal subgroup comparisons would require a larger sample. Sixth, the survey relied on self-report and was therefore subject to social desirability and recall biases and to the common method bias noted above. Finally, this study is descriptive and correlational; causal inferences about the effects of eHealth literacy on patient outcomes cannot be drawn and will require longitudinal or interventional designs.

### 4.8. Future Research

Three lines of future work are warranted. First, prospective cohort studies linking eHealth literacy to objective, prospectively measured outcomes such as medication adherence (e.g., proportion of days covered), disease-modifying therapy persistence, unplanned healthcare utilisation, and patient-reported quality of life would strengthen the causal evidence base, ideally testing eHealth literacy as a predictor while examining age, education, and disease severity as moderators and appraisal behaviour as a mediator. Second, larger studies are needed to permit formal subgroup comparisons between patients and carers; our patient-only sensitivity analysis (*n* = 62–79) was exploratory and underpowered for formal interaction testing, and the equivalence of patient and carer responses should not be assumed without confirmatory evidence from a study designed to detect subgroup differences. Third, controlled evaluations of clinician-mediated signposting interventions would directly test whether the strong patient preference for clinician-endorsed information translates into improved information quality and decision-making. Fourth, the design and pilot evaluation of a transparently assessed MS information resource, incorporating the features endorsed by participants here (Figure 4), would represent a logical next step and a feasible proof of concept for the broader eHealth literacy agenda in chronic disease.

## 5. Conclusions

Among this engaged UK sample, people living with MS were near-universal users of the Internet for health information but encountered substantial difficulty in independently evaluating the quality of what they found; whether this pattern holds in less digitally engaged subgroups requires confirmation in more representative samples. Clinician recommendation is the most powerful cue for perceived quality, and a transparently assessed, curated information resource, with visible quality indicators, dedicated MS and medicine search functionalities, and full accessibility, was endorsed by the subgroup of participants who reached these exploratory items and represents a promising but not yet established direction. The strong intercorrelation of confidence, perceived importance, and active checking was observed in the combined sample of patients and carers; because a patient-only sensitivity analysis was not performed, it remains untested whether these relationships are driven by carer-specific responses. Targeted eHealth literacy interventions, particularly those mediated by clinicians and specialist nurses, nonetheless remain a promising target for future evaluation; the present cross-sectional data cannot establish their effectiveness. The integration of such interventions into routine MS care, alongside the development of charity- or NHS-endorsed curated resources, represents a practical path toward higher-quality online information for this growing patient population.

## Figures and Tables

**Table 1:** Demographic and background characteristics of participants (*n* = 152).

Variable	Category	*n*	%
Respondent type	Person with MS	128	84.2
	Carer	22	14.5
	Missing	2	1.3
Gender	Male	45	31.7
	Female	97	68.3
Age group (years)	18–35	22	15.4
	36–45	14	9.8
	46–55	53	37.0
	56–60	34	23.8
	>60	20	14.0
Education	Postgraduate (e.g., MSc/PhD)	39	27.3
	Undergraduate (e.g., BSc)	43	30.1
	A-level or equivalent	37	25.9
	Secondary education only	21	14.7
MS subtype	Relapsing–remitting (RRMS)	54	37.8
	Secondary progressive (SPMS)	49	34.3
	Primary progressive (PPMS)	35	24.5
	Unknown	5	3.5

Note: Percentages are calculated against the valid denominator for each variable; missing values are excluded except for respondent type, where the two missing responses are explicitly shown. Percentages are based on all respondents (*n* = 152) and include carers unless otherwise noted. The sample comprised 128 people with MS and 22 carers, with the respondent type missing for 2 participants. All variables in this table, including MS subtype and educational level, are reported for the combined sample of people with MS and carers.

**Table 2:** Spearman rank-order correlations between key study constructs, with pairwise *n*, 95% confidence intervals, and FDR-adjusted *p*-values.

Construct Pair	ρ	*n*	95% CI	*p*	*p* (FDR)
Confidence × Perceived importance	0.869 †	73	[0.78, 0.92]	<0.001	<0.001
Confidence × Vigilance in checking	0.677	73	[0.51, 0.79]	<0.001	<0.001
Confidence × Readiness of medicine information	0.553	73	[0.36, 0.70]	<0.001	<0.001
Confidence × Total MS symptoms	0.731	73	[0.58, 0.83]	<0.001	<0.001
Confidence × Total treatments received	0.273	73	[0.04, 0.48]	0.019	0.019
Confidence × PIL understanding	0.583	73	[0.39, 0.73]	<0.001	<0.001
Perceived importance × Vigilance in checking	0.641	73	[0.46, 0.77]	<0.001	<0.001
Perceived importance × Readiness of medicine information	0.636	73	[0.46, 0.77]	<0.001	<0.001
Perceived importance × Total MS symptoms	0.722	73	[0.57, 0.83]	<0.001	<0.001
Perceived importance × PIL understanding	0.536	73	[0.33, 0.69]	<0.001	<0.001
Readiness of medicine information × Total MS symptoms	0.437	94	[0.25, 0.59]	<0.001	<0.001
Readiness of medicine information × PIL understanding	−0.279	94	[−0.46, −0.08]	0.006	0.007

Note. ρ = Spearman’s rank-order correlation coefficient. *n* = pairwise-complete cases: the medicine–confidence and medicine–importance items were answered by 73 participants and the readiness items by 94. The 95% confidence intervals were derived by Fisher z-transformation using the Bonett–Wright standard error for Spearman’s ρ. *p*-values are two-tailed; *p* (FDR) = Benjamini–Hochberg false discovery rate-adjusted *p* across the 12 tests. All associations remained significant after FDR adjustment. † The confidence–perceived importance association (ρ = 0.869) exceeds the ρ > 0.80 threshold and is interpreted as empirically overlapping rather than fully distinct (see Section 4.5). All correlations in this table were computed on the combined sample of people with MS and carers. A patient-only sensitivity analysis (reported in Supplementary Table S1) demonstrated highly consistent findings: the direction, magnitude, and statistical significance of all associations were preserved in the patient-only subsample, with correlation coefficients differing by no more than 0.02–0.03 from the combined-sample estimates.

## Data Availability

The datasets generated and analysed during the current study are not publicly available due to copyright and ownership considerations and because portions of the data are reserved for additional analysis in a related manuscript. The datasets are available from the corresponding author on reasonable request.

## Supplementary Materials

The following supporting information can be downloaded at https://www.mdpi.com/article/10.3390/healthcare14182927/s1. Table S1: Patient-only sensitivity analysis: Spearman rank-order correlations between key study constructs in the subsample restricted to individuals with MS (*n* = 62 for medicines-confidence/importance scales; *n* = 79 for readiness scales).

## Abbreviations

MS: Multiple Sclerosis; UK: United Kingdom; RRMS: Relapsing–Remitting MS; SPMS: Secondary Progressive MS; PPMS: Primary Progressive MS; DMT: Disease-Modifying Therapy; GP: General Practitioner; NHS: National Health Service; eHEALS: eHealth Literacy Scale; CHERRIES: Checklist for Reporting Results of Internet E-Surveys; STROBE: Strengthening the Reporting of Observational Studies in Epidemiology.
