# Supplementary material for: Trust, Information Appraisal, and Health Communication Behaviours Among People Living with Multiple Sclerosis
Source: Healthcare (Basel). 2026 Sep 9;14(18):2927. doi: 10.3390/healthcare14182927 (PMC13606548; doi:10.3390/healthcare14182927)
Supplement: Supplementary file 1 [file healthcare-14-02927-s001.zip › healthcare-4387548-supplementary.pdf]

New Supplementary Table S1

Supplementary Table S1. Patient-only sensitivity analysis: Spearman rank-order correlations between key study constructs in the subsample restricted to individuals with MS (n = 62 for medicines-confidence/importance scales; n = 79 for readiness scales).

**Construct Pair**	**ϱ**	**n**	**95% CI**	**p**	**p (FDR)**
Confidence × Perceived importance	0.891	62	[0.80, 0.94]	<0.001	<0.001
Confidence × Vigilance in checking	0.694	62	[0.53, 0.81]	<0.001	<0.001
Confidence × Readiness of medicines information	0.571	62	[0.36, 0.73]	<0.001	<0.001
Confidence × Total MS symptoms	0.748	62	[0.60, 0.85]	<0.001	<0.001
Confidence × Total treatments received	0.289	62	[0.05, 0.50]	0.023	0.023
Confidence × PIL understanding	0.601	62	[0.40, 0.75]	<0.001	<0.001
Perceived importance × Vigilance in checking	0.659	62	[0.48, 0.79]	<0.001	<0.001
Perceived importance × Readiness of medicines information	0.643	62	[0.46, 0.77]	<0.001	<0.001
Perceived importance × Total MS symptoms	0.739	62	[0.58, 0.84]	<0.001	<0.001
Perceived importance × PIL understanding	0.557	62	[0.35, 0.71]	<0.001	<0.001
Readiness of medicines information × Total MS symptoms	0.429	79	[0.23, 0.59]	<0.001	<0.001
Readiness of medicines information × PIL understanding	-0.282	79	[-0.47, -0.07]	0.012	0.012

Note: ρ = Spearman's rank-order correlation coefficient. n = pairwise-complete cases. 95% confidence intervals were derived by Fisher z-transformation using the Bonett-Wright standard error for Spearman's ρ. p-values are two-tailed; p (FDR) = Benjamini-Hochberg false-discovery-rate-adjusted p across the 12 tests. All associations remained significant after FDR adjustment. These results are consistent with those observed in the combined sample (Table 2), with correlation coefficients differing by no more than 0.02-0.03.
